# Efficacy and Safety of Low-Dose Cyclosporine with Everolimus and Steroids in *de novo* Heart Transplant Patients: A Multicentre, Randomized Trial

**DOI:** 10.1155/2011/535983

**Published:** 2011-09-13

**Authors:** Andreas Zuckermann, Shoei-Shen Wang, Heather Ross, Maria Frigerio, Howard J. Eisen, Christoph Bara, Daniel Hoefer, Maurizio Cotrufo, Gaohong Dong, Guido Junge, Anne M. Keogh

**Affiliations:** ^1^Department of Cardiothoracic Surgery, University of Vienna, 1090 Vienna, Austria; ^2^Division of Cardiovascular Surgery, National Taiwan University Hospital, Taipei 100, Taiwan; ^3^Department of Cardiology, Toronto General Hospital, Toronto, ON, Canada M562C4; ^4^Divisione di Cardiologia, Azienda Ospedale Niguarda Ca' Granda, 20162 Milano, Italy; ^5^Division of Cardiology, Drexel University College of Medicine, Philadelphia, PA 19102, USA; ^6^Klinik für Thorax-, Herz- und Gefäßchirurgie, Medizinische Hochschule Hannover, 30625 Hannover, Germany; ^7^Department of Cardiac Surgery, Innsbruck Medical University, 6020 Innsbruck, Austria; ^8^Dipartimento di Scienze Cardio-Toraciche e Respiratorie Sezione di Cardiologia Pediatrica, Azienda Ospedaliera Monaldi Cotugno Il Università di Napoli, 80122 Napoli, Italy; ^9^Novartis Pharmaceuticals Corporation, East Hanover, NJ 07936-1016, USA; ^10^Novartis Pharma AG, 4002 Basel, Switzerland; ^11^Heart Lung Transplant Unit, St. Vincent's Hospital, Sydney, NSW 1340, Australia; ^12^Department of Cardiothoracic Surgery, Medical University of Vienna, Waehringer Guertel 18-20, 1090 Vienna, Austria

## Abstract

A six-month, multicenter, randomized, open-label study was undertaken to determine whether renal function is improved using reduced-exposure cyclosporine (CsA) versus standard-exposure CsA in 199 *de novo* heart transplant patients receiving everolimus and steroids ± induction therapy. Mean C_2_ levels were at the low end of the target range in standard-exposure patients (*n* = 100) and exceeded target range in reduced-exposure patients (*n* = 99) throughout the study. Mean serum creatinine at Month 6 (the primary endpoint) was 141.0 ± 53.1 *μ*mol/L in standard-exposure patients versus 130.1 ± 53.7 *μ*mol/L in reduced-exposure patients (*P* = 0.093). The incidence of biopsy-proven acute rejection ≥3A at Month 6 was 21.0% (21/100) in the standard-exposure group and 16.2% (16/99) in the reduced-exposure group (n.s.). Adverse events and infections were similar between treatment groups. Thus, everolimus with reduced-exposure CsA resulted in comparable efficacy compared to standard-exposure CsA. No renal function benefits were demonstrated; that is possibly related to poor adherence to reduced CsA exposure.

## 1. Introduction

 One-year survival following cardiac transplantation has risen to approximately 85%, but long-term graft loss remains a significant problem with life expectancy 12 years after transplantation remaining at only 50% [1]. Late-term complications include renal dysfunction, malignancy, and cardiac allograft vasculopathy (CAV) [1–3]. In a randomized trial of everolimus versus azathioprine with standard-exposure cyclosporine (CsA) and steroids in de novo heart transplant recipients, use of everolimus significantly reduced coronary artery intimal proliferation, assessed by intravascular ultrasound, and the incidence of CAV up to 24 months [4, 5]. While everolimus is not associated with direct renal toxicity [6], it can potentiate CsA-related nephrotoxicity by P450 inhibition of CsA metabolism [7], and serum creatinine levels were higher among patients receiving everolimus in this study. This was found to be due to the use of fixed-dose administration of everolimus, instead of concentration-controlled dosing, and because CsA was given at a standard level of exposure [4]. Accordingly, everolimus dosing is now based on blood concentration and reduced CsA dosing is recommended in the maintenance phase to preserve renal function in cardiac transplant recipients receiving everolimus [8]. The efficacy and safety of everolimus with CNI minimization in maintenance thoracic transplant recipients has been demonstrated in the prospective NOCTET study [9] as well as in a single-arm pilot study [10, 11] and two single-centre trials [12, 13]. To date, however, no prospective study has attempted to determine the optimal CsA exposure in *de novo* heart transplant patients receiving everolimus. Moreover, no trial has assessed CsA exposure in *de novo* everolimus-treated heart transplant recipients based on C_2_ monitoring, which has been shown to lead to clinical benefits versus conventional C_0_ (trough level) monitoring [14–20]. 

A six-month, multicentre, randomized, open-label study was undertaken to determine whether renal function is improved in *de novo* heart transplant patients receiving reduced-exposure CsA versus standard-exposure CsA, based on C_2_ monitoring, when administered in combination with everolimus and steroids, with or without induction therapy. 

## 2. Materials and Methods

### 2.1. Study Design

This was a six-month, multicentre, prospective, randomized, open-label study (clintrials.gov reference number NCT00098007) comparing renal function (serum creatinine) in de novo heart transplant patients receiving everolimus with either reduced-dose (RD) or standard-dose (ST) CsA microemulsion (CsA-ME). Randomization took place within 72 hours of transplantation on a 1 : 1 basis using unique patient identification numbers assigned centrally. The study was conducted in accordance with the ICH Harmonized Tripartite Guidelines for Good Clinical Practice and the Declaration of Helsinki after obtaining approval from the Institutional Review Board at each centre and written informed consent from all patients. 

### 2.2. Patients

The population comprised patients aged 18–65 years receiving a primary heart transplant. Key exclusion criteria were donor >60 years of age or with obvious coronary disease or known heart disease, cold ischaemia time >6 hours, receipt of a multiorgan transplant or any previous organ transplant, serum creatinine level >250 *μ*mol/L, platelet count ≤50,000/mm^3^ or white blood cell count of ≤2,500/mm^3^, panel reactive antibodies ≥25%, severe hypercholesterolemia (≥9 mmol/L), or hypertriglyceridemia (≥8.5 mmol/L). 

### 2.3. Immunosuppression and Concomitant Medication

Centres were permitted to use antithymocyte globulin (ATG) or interleukin (IL)-2 receptor antagonist induction consistently for all patients at that centre. CsA-ME (Neoral, Novartis, Basel, Switzerland) was initiated at ≤12 mg/kg/day, except at centres using induction therapy, where local practice for CsA-ME introduction was followed. The dose was subsequently adjusted to maintain a predefined target C_2_ range based on previous studies of C_2_-based CsA monitoring in heart transplant recipients (19, 20). The CsA C_2_ target range was 1000–1400 ng/mL for all patients during the first two months after transplant, after which CsA targets were lowered according to the randomized groups: ST group 800-1200 ng/mL during Months 3–5 and 600–1000 ng/mL during Month 6; RD group 600–800 ng/mL during Month 3, 400-600 ng/mL during Months 4-5 and 300–500 ng/mL during Month 6. The CsA C_2_ levels were determined at study days 2, 3, 5, 8, 15, 22, and 30 during the first month and monthly thereafter. Everolimus (Certican, Novartis Pharma AG, Basel, Switzerland) was initiated within 72 hours after transplant, at an initial dose of 0.75 mg b.i.d., titrated after Day 5. Everolimus trough (C_0_) level was recorded on Day 5 post-randomization, after which the dose was titrated to achieve a C_0_ level in the range 3–8 ng/mL. Cytomegalovirus (CMV) prophylaxis was to be applied as per local center practice.

### 2.4. Primary Endpoint and Statistical Analysis

The primary endpoint was renal function at six months after transplant, as measured by serum creatinine. This was compared between treatment groups using the *t*-test (0.05 one-sided significance level). A sample size of 200 patients (100 per treatment arm) was estimated to have 82% power to detect a difference of 22 *μ*mol/L in mean serum creatinine level at 6 months post-transplant assuming that mean creatinine would be 177 ± 60 *μ*mol/L in the ST group and 155 ± 60 *μ*mol/L in the RD group (*t*-test with 0.05 one-sided significance level). Three preplanned supportive analyses were performed on renal function for the ITT population. One supportive analysis was based on the “on-treatment” Month 6 renal function value. An on-treatment observation was defined as a measurement obtained ≤2 days after discontinuation of randomized study medication. The other two supportive analyses were performed for all patients alive at the Month 6 visit based on the following imputation methods for missing renal function value: (i) last observation carried forward (LOCF) and (ii) multiple imputation. Post hoc analyses were performed in the patients who remained within CsA C_2_ target ranges throughout the study. Between-group differences in efficacy event rates were compared using the two-sided *z*-test. 

## 3. Results

### 3.1. Patients

**Figure 1:**
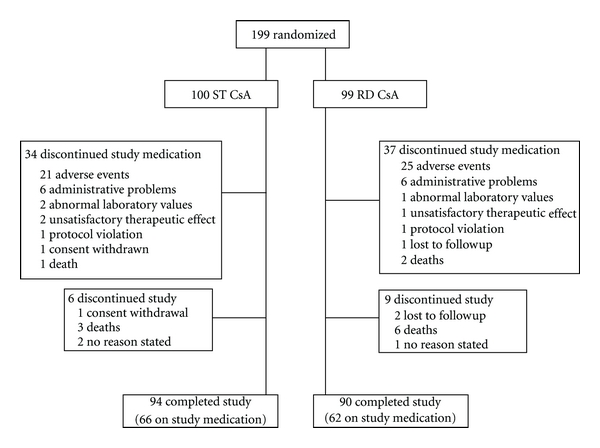
Patient disposition.

In total, 199 patients were enrolled, randomized, and formed the ITT (intent-to-treat) and safety populations, of whom 184 patients completed the six-month study, 128 on study medication (Figure 1). Patient characteristics were similar in the RD group (*n* = 99) and the ST group (*n* = 100) except of a lower mean cold ischemia time in the RD group (RD: 2.8 ± 1.1 h  versus ST: 3.2 ± 1.2 h; *P* = 0.027) (Table 1). 

### 3.2. Immunosuppression and Concomitant Medication

**Figure 2:**
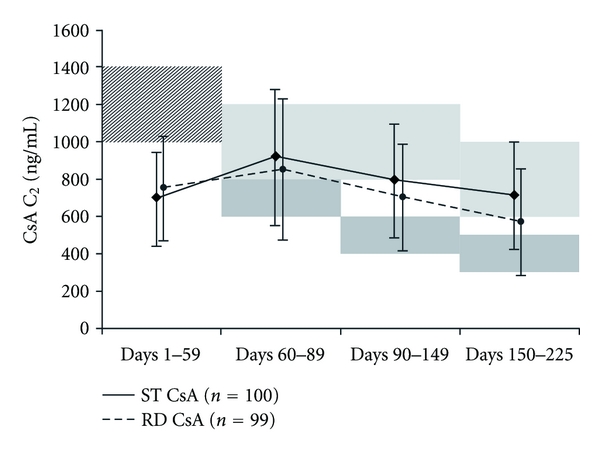
CsA C_2_ level during the six-month study. Shaded areas indicate target C_2_ ranges (hatched area, shared target to Day 59; dark area, reduced-CsA; light area, standard-CsA). Values are shown as mean±SD (central laboratory results).

Mean everolimus trough levels remained within the target range (3–8 ng/mL) in both cohorts at all study visits. During Months 1-2, mean CsA C_2_ levels were markedly below target in both groups and, indeed, higher in the RD group than the ST group (RD 742 ± 272 ng/mL; ST 693 ± 244 ng/mL). Subsequently, the mean C_2_ levels exceeded target range in the RD patients throughout the study and were near the lower end of the target range in the ST cohort (Figure 2). Mean steroid dose over the six-month study was similar in both groups (Table 2, *P* = 0.23), as was the percentage of patients that received induction therapy. Use of concomitant medication that could potentially influence renal function, including angiotensin converting enzyme inhibitors and aldosterone receptor blockers, was similar between treatment groups (data not shown). CMV prophylaxis was used in 42.4% of patients in the RD group and in 49% of patients in the ST cohort.

### 3.3. Renal Function

**Figure 3:**
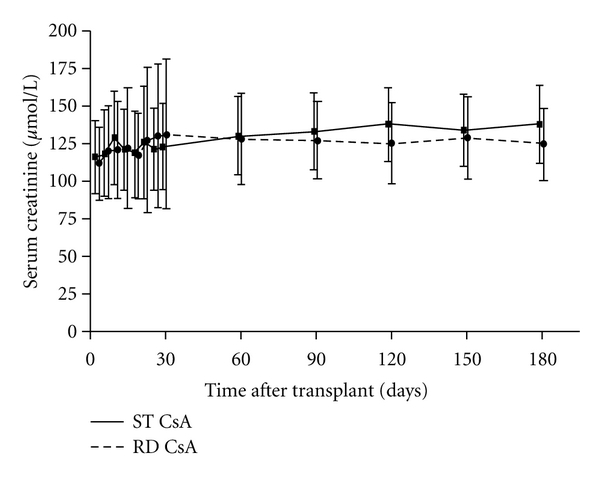
Serum creatinine during the six-month study (ITT population). Values shown are mean ± SD.

Mean serum creatinine at baseline was 111.6 ± 46.4 *μ*mol/L in the RD arm and 116.0 ± 47.0 *μ*mol/L in the ST group. At Month 6, mean serum creatinine was lower in the RD group than in the ST cohort but the difference was not significant (130.1 ± 53.7 *μ*mol/L versus 141.0 ± 53.1 *μ*mol/L,   *P* = 0.093) (Figure 3). The mean increase in serum creatinine from baseline to Month 6 was 15.9 ± 52.7 *μ*mol/L in the RD group compared to 27.9 ± 64.0 *μ*mol/L in the ST arm (*P* = 0.102). Predefined supportive analyses to account for missing Month 6 creatinine measurements showed a significantly lower mean creatinine level at Month 6 in the RD versus the ST group (LOCF: RD 127.3 *μ*mol/L, ST 145.9 *μ*mol/L, *P* = 0.023; multiple imputation, RD: 127.9 *μ*mol/L, ST 143.8 *μ*mol/L, *P* = 0.027). 

 Mean eGFR (MDRD) at Month 6 was similar in the RD group (59.0 ± 23.2 mL/min /1.73 m^2^) and ST arm (59.5 ± 48.2 mL/min /1.73 m^2^). The decline in eGFR from baseline was numerically lower in the RD group, but this change was not statistically significant (−10.4 ± 23.2 versus −12.9 ± 33.8, *P* = 0.298).

A post hoc analysis was performed to analyze the change in renal function from baseline to Month 6 for patients who received CsA reductions as required by protocol (RD, *n* = 20; ST, *n* = 32). Whereas the mean change in creatinine from baseline to Month 6 was 5.5 ± 45.1 *μ*mol/L in the RD group, patients in the ST group had a mean increase in creatinine of 31.4 ± 57.7 *μ*mol/L (*P* = 0.047). The corresponding change in eGFR was −8.9 ± 18.6 mL/min /1.73 m^2^ in the RD group and −14.0 ± 36.3 mL/min /1.73 m^2^ in the ST cohort (*P* = 0.267).

### 3.4. Efficacy

There was no significant between-group difference in the incidence of the composite endpoint at Month 6, or in any individual efficacy component (Table 3). Across the total population, the incidence of BPAR ≥3 A was 18.6% (37/199). The incidence of BPAR ≥3 A was numerically lower in the RD group among patients who received induction (17/81 ST [21.0%] versus 12/81 RD [14.8%], n.s.) and similar with both CsA regimens in the no-induction group (4/19 ST [21.1%] and 4/18 RD [22.2%]). Three patients died in the ST group (2 multiorgan failure and 1 unknown cause with no suspected relation to study drug) and six patients in the RD group (2 sepsis, 1 encephalitis, 1 primary graft failure, 1 unknown cause with no suspected relation to study drug, and 1 unknown cause in a patient experiencing sudden death in whom autopsy revealed an aortic anastomosis leak that the investigator suspected to have a relationship to study drug). 

### 3.5. Safety

The type and incidence of adverse events and infections was similar between treatment groups (Table 4). Adverse events or infections with a suspected relation to study drug were reported in 59 ST patients (59.0%) and 58 RD patients (58.6%). Serious adverse events occurred in 57 ST patients (57.0%) and in 60 RD patients (60.6%). CMV infection (defined as positive antigenemia and/or PCR and/or seroconversion without signs and/or symptoms) occurred in three ST patients (3.0%) and seven RD patients (7.1%). Renal failure and acute renal failure were reported as an adverse event in 25 ST patients (25.0%) and 22 RD patients (22.2%). In >60% of cases renal failure was diagnosed during the first two weeks after transplantation and resolved within four weeks. Twelve (12%) patients in the ST group and thirteen (13.1%) recipients in the RD group required temporary dialysis. Five (5%) ST patients and seven (7.1%) RD patients discontinued study prematurely due to renal failure/acute renal failure. Wound healing complications relating to the surgical intervention were reported as serious adverse events in eight (8.0%) and ten (9.9%) patients in the ST and RD groups, respectively (n.s.). One patient in the study experienced an episode of pneumonitis, possibly induced by everolimus. There were no marked differences in hematological or laboratory parameters between treatment groups. From baseline to Month 6, the mean change in total cholesterol, LDL-cholesterol, HDL-cholesterol, and triglyceride concentration in the ST and RD groups was 2.16 mmol/L and 2.42 mmol/L (*P* = 0.25), 1.17 mmol/L and 1.33 mmol/L (*P* = 0.31), 0.61 mmol/L and 0.55 mmol/L (*P* = 0.77), and 0.76 mmol/L and 1.62 mmol/L (*P* = 0.06), respectively. Twenty-one patients (21.0%) and 25 patients (25.3%) in the ST and RD groups, respectively, discontinued study medication due to adverse events. Haematological disorders (mostly leukopenia, thrombocytopenia, and anaemia) leading to discontinuation were observed in two ST patients (2.0%) and three RD patients (3.0%). 

## 4. Discussion

The well-established nephrotoxicity associated with calcineurin inhibitors has prompted the exploration of immunosuppressive regimens that maintain low rejection rates while minimizing deterioration of renal function. The current study was undertaken to investigate whether a reduction in CsA exposure, as monitored by C_2_ levels, in combination with everolimus and corticosteroids would help to preserve renal function following heart transplantation without compromising protection against acute rejection compared with standard CsA exposure. Throughout the study, however, there was poor adherence to planned CsA exposure levels such that although efficacy was indeed similar between treatment groups there was no significant difference in creatinine values at Month 6—the primary endpoint—or in mean eGFR. 

At all time points, fewer than half the patients were within CsA C_2_ target range: in fact, the mean C_2_ level was higher in Month 3 than during Months 1-2 despite a planned decrease in C_2_ concentration, while at Month 6, the RD group had only a 20% reduction in CsA exposure compared to the ST group. Reasons for nonadherence to protocol-specified target ranges lied in the investigator inexperience with CsA C_2_ monitoring, the concern of CsA underexposure and related rejection risk for the RD arm especially during the first month, and finally the concern of CsA overexposure and previously described renal toxicity [4] in the ST arm. In heart transplant recipients, the potential penalty of graft loss and death in the setting of rejection is greater than recurrent dialysis in renal transplant recipients with graft loss, leading to greater caution about lowering immunosuppression. Moreover, if there were signs of rejection on the latest endomyocardial biopsy, then the protocol stipulated that the CsA dose was not to be reduced after Month 2. With ~40% of patients having Grade 1A rejection reported, this frequently prevented lowering of CsA and hence lower exposure levels. Interestingly, in the small number of patients whose CsA C_2_ remained within target range, post hoc analysis showed a smaller increase in serum creatinine from baseline and improved eGFR in the RD group versus the ST arm. The A2411 de novo heart transplant recipient study compared everolimus with reduced exposure CsA to MMF with standard exposure CsA. The study did not achieve demonstration of noninferior renal function with everolimus at Month 6 possibly related to a higher than targeted CsA exposure in the everolimus arm [21]. Randomized, multicenter studies recruiting large patient numbers across different study sites and countries are important to obtain robust data on the efficacy and safety of immunosuppressive regimens; however, adherence to protocol specifications can vary possibly reflecting differences in experience and confidence into the studied treatment regimen.

A second drawback was the six-month duration of the study; with hindsight, a longer followup would have been helpful. The six-month study period was selected based on evidence from the large, prospective trial undertaken by Eisen et al., in which significant differences in renal function were observed within the first six months (indeed, by the end of Month 1) in patients randomized to everolimus with standard-dose CsA versus azathioprine-treated controls [4]. Serum creatinine concentrations at Month 12 from 18 patients who continued the assigned regimen in an umbrella protocol were reviewed. Mean serum creatinine was 116.1 ± 48.0 *μ*mol/L among seven patients in the RD arm and 153.4 ± 29.2 *μ*mol/L among 11 patients randomized to the ST arm. While this suggests benefit of the RD regimen could be maintained at Month 12, the small patient numbers mean that this can only be speculation. 

Although efficacy was good, as indicated by a low rate of BPAR ≥3 A, and was not compromised in either the RD group or among patients without induction therapy, the overlap in CsA exposure between treatment groups does not allow us to confirm whether reduced CsA exposure provides a similar rate of rejection to a standard CsA regimen. Though cross-study comparisons need to be interpreted with caution, it is noteworthy that rejection rates in the RD arm of this study were comparable to rates of BPAR ≥3 A seen for everolimus with standard CsA exposure in the earlier study B253 (27.8% and 19.0% for everolimus 1.5 mg and 3 mg, resp.).

In conclusion, everolimus with CsA C_2_ monitoring and corticosteroids resulted in a low rate of BPAR ≥3 A with good graft survival at six months and acceptable renal function preservation in *de novo* heart transplant recipients. However, poor adherence to the planned CsA exposure ranges meant that the potential benefit of CsA reduction could not be evaluated. Regarding the design of future studies, the simultaneous introduction of two novel procedures—in this case CsA lowering and use of C_2_ monitoring—into a clinical study protocol increases the risk of poor adherence to the protocol. Future protocols should consider measures to improve CsA exposure adherence. Further randomized studies are required to clarify if renal function in *de novo* heart transplant recipients can be preserved using a combination of everolimus and reduced-exposure CsA.

## Figures and Tables

**Table 1:** Demographics and baseline characteristics. Continuous variables are shown as mean ± SD.

	RD CsA (*n* = 99)	ST CsA (*n* = 100)
Recipient age (years)	49.8 ± 11.77	49.4 ± 10.44
Female recipient	25 (25.3%)	22 (22.0%)
White recipient	79 (79.8%)	77 (77.0%)
End stage disease leading to transplantation		
Cardiomyopathy	49 (49.5%)	55 (55.0%)
Coronary artery disease	32 (32.3%)	30 (30.0%)
Other	18 (18.2%)	15 (15.0%)
Ventricular assist device	12 (12.1%)	8 (8.0%)
Panel reactive antibodies (%)		
0%–10%	84 (84.5%)	87 (87.0%)
>10%	1 (1.0%)	0
Missing	14 (14.1%)	13 (13.0%)
Donor age (years)	35.3±13.9	35.5±11.9
Female donor	24 (24.2%)	35 (35.0%)
Cytomegalovirus D+/R−	23 (23.2%)	17 (17.0%)
Cold ischaemia time (hours) [mean ± SD]	2.8 ± 1.1	3.2 ± 1.2
Diabetes	31 (31.3%)	31 (31.0%)
Hypertension	45 (45.5%)	53 (53.0%)

**Table 2:** Immunosuppression. Continuous variables are shown as mean ± SD.

	RD CsA (*n* = 99)	ST CsA (*n* = 100)
Everolimus dose (mg/day)		
Month 1	1.3 ± 0.5	1.4 ± 0.6
Month 6	1.4 ± 0.6	1.3 ± 0.5
Everolimus trough concentration (ng/mL)		
Month 1	5.8 ± 3.6	5.8 ± 2.5
Month 6	4.8 ± 1.7	5.3 ± 2.3
Cyclosporine dose (mg/kg/day)		
Month 1	3.8 ± 1.3	4.0 ± 1.4
Month 6	2.5 ± 1.0	2.8 ± 0.8
Cyclosporine trough concentration (ng/mL)		
Months 1-2	195 ± 78	209 ± 87
Month 6	120 ± 63	154 ± 68
Cyclosporine C_2_ concentration (ng/mL)		
Months 1-2	742 ± 272	693 ± 244
Month 6	566 ± 278	707 ± 284
Steroid dose^a^	0.50 ± 1.40	0.32 ± 0.25
Induction therapy		
Antithymocyte globulin	60 (60.6%)	61 (61.0%)
Interleukin-2 receptor antagonist	21 (21.2%)	20 (20.0%)

^a^ Mean dose during Months 0–6.

**Table 3:** Incidence of efficacy events at Month 6. HDC, haemodynamic compromise.

	RD CsA (*n* = 99)	ST CsA (*n* = 100)
Composite efficacy failure (BPAR ≥3 A, acute rejection associated with HDC, death, graft loss/retransplant or lost to followup)	26 (26.3%)	25 (25.0%)
BPAR ≥3 A	16 (16.2%)	21 (21.0%)
Acute rejection associated with HDC	3 (3.0%)	4 (4.0%)
Graft loss	1 (1.0%)	1 (1.0%)
Death	6 (6.1%)	3 (3.0%)

All differences were non-significant.

**Table 4:** Incidence of infections and adverse events of interest.

	RD CsA (*n* = 99)	ST CsA (*n* = 100)
Any adverse event, or infection or serious adverse event	99 (100.0%)	100 (100.0%)
Infection		
Any	52 (52.5%)	47 (47.0%)
Bacterial	29 (29.3%)	18 (18.0%)
Fungal	2 (2.0%)	7 (7.0%)
Viral	13 (13.1%)	7 (7.0%)
CMV	7 (7.1%)	3 (3.0%)
Other	5 (5.1%)	9 (9.0%)
Unknown	26 (26.3%)	27 (27.0%)
Anaemia	21 (21.2%)	16 (16.0%)
Thrombocytopenia	5 (5.1%)	6 (6.0%)
Leukopenia	12 (12.1%)	7 (7.0%)
Incision-site related wound healing complications^1^	10 (9.9%)	8 (8.0%)
Cardiac tamponade^2^	7 (6.9%)	5 (5.0%)
Effusion-related complications^3^	5 (5%)	8 (8.0%)

^1^Wound healing complications associated with a surgical procedure that were reported as serious adverse events.

^2^Three more cardiac tamponades occurred after iatrogenic myocardial perforation during biopsy procedure (2 ST and 1 RD).

^3^Pleural and pericardial effusions that were reported as serious adverse events.

## Appendix

### The RAD A2403 Study Group

*Australia*: Anne M Keogh and Deborah Meyers. *Austria: *Andreas Zuckermann and Guenther Laufer. *Brazil:* Edimar Bocchi and Maria C Moreira. *Canada:* Heather Ross and Andrew Ignaszewski. *Germany:* Christoph Bara and Stephan Hirt, *Italy: *Maria Frigerio and Maurizio Cotrufo. *Poland: *Malgorzata Sobieszczanska and Marian Zembala. *Spain: *Eulalia Roig Minguell, Marisa Crespo, and Ernesto Lage. *Taiwan: *Shoei-Sheng Wang. *USA: *Howard J. Eisen, Leslie W. Miller, Alfred J. Tector, Mark W. Weston, Gary M. Felker, Mark J. Zucker, Lee Goldberg, Frank H. Smart, and Reynold Delgado.
